# The effects of anti-PD-1 therapy on programmed death-ligand 1 expression and glucose metabolism of normal organs in patients with advanced non-small cell lung cancer

**DOI:** 10.1093/bjro/tzaf010

**Published:** 2025-05-13

**Authors:** Matthew Severyn, Eunice H L Lee, Gitasha Chand, Jessica Johnson, Damion Bailey, Kathryn Adamson, Vicky Goh, Daniel Johnathan Hughes, Gary J R Cook

**Affiliations:** GKT School of Medicine: King’s College London Faculty of Life Sciences & Medicine, London, United Kingdom; GKT School of Medicine: King’s College London Faculty of Life Sciences & Medicine, London, United Kingdom; Department of Cancer Imaging, School of Biomedical Engineering and Imaging Sciences, King’s College London, London, SE1 7EH, United Kingdom; Department of Nuclear Medicine, Guy’s and St Thomas’ NHS Foundation Trust, London, SE1 9RT, United Kingdom; Department of Nuclear Medicine, Guy’s and St Thomas’ NHS Foundation Trust, London, SE1 9RT, United Kingdom; Department of Nuclear Medicine, Guy’s and St Thomas’ NHS Foundation Trust, London, SE1 9RT, United Kingdom; Department of Cancer Imaging, School of Biomedical Engineering and Imaging Sciences, King’s College London, London, SE1 7EH, United Kingdom; Department of Cancer Imaging, School of Biomedical Engineering and Imaging Sciences, King’s College London, London, SE1 7EH, United Kingdom; Department of Cancer Imaging, School of Biomedical Engineering and Imaging Sciences, King’s College London, London, SE1 7EH, United Kingdom; King’s College London & Guy’s and St Thomas’ PET Centre, Guy’s and St Thomas’ NHS Foundation Trust, London, SE1 7EH, United Kingdom

**Keywords:** [99mTc]NM-01, non-small cell lung cancer, PET/CT imaging

## Abstract

**Objectives:**

To investigate how anti-PD-1 treatment affects both Programmed Death-Ligand 1 (PD-L1) expression and glucose metabolism within normal tissues of advanced non-small cell lung cancer (NSCLC) patients using a dual SPECT/CT and PET/CT imaging approach.

**Methods:**

Ten advanced NSCLC patients (NCT04436406) undergoing anti-PD-1 therapy ± chemotherapy underwent imaging at baseline and 9 weeks. PD-L1 expression was measured using [^99m^Tc]-labelled single-domain PD-L1 antibody single-photon emission computed tomography/computed tomography ([^99m^Tc]NM-01 SPECT/CT). Glucose uptake was measured using [^18^F]-Fluorodeoxyglucose positron emission tomography/computed tomography ([^18^F]FDG PET/CT). Two independent observers marked regions of interest across normal organs (liver, lung, spleen, bone marrow, muscle, kidney, pancreas, left ventricular myocardium, and blood pool) to determine maximum and mean standardized uptake values (SUV) at both time points. Observer agreement was measured with an intraclass correlation coefficient (ICC).

**Results:**

No significant changes in SUVs, indicating PD-L1 expression and glucose metabolism, were detected in normal organs after 9 weeks of treatment (all *P* > .05). No patients developed immune-related adverse events (irAEs) during the study period. Observer measurements showed excellent consistency with an ICC of 0.99 (95% confidence interval 0.99-0.99).

**Conclusions:**

Our study showed stable PD-L1 expression and glucose metabolism within normal organs in advanced NSCLC patients treated with anti-PD-1 therapy ± chemotherapy. Interobserver reliability between observers was excellent. Additional studies with larger patient groups and a specific focus on irAE cases are needed.

**Advances in knowledge:**

Through a dual-modality molecular imaging approach, this research provides novel insight into anti-PD-1 therapy’s effects on PD-L1 expression and glucose metabolism in normal organs of NSCLC patients, demonstrating that these parameters remain stable post-treatment.

## Introduction

Immune checkpoint inhibitors, including those targeting the programmed cell death protein 1 (PD-1) and programmed death ligand 1 (PD-L1) pathway, have revolutionized the treatment approach of many cancers, including non-small cell lung cancer (NSCLC). Under normal physiological conditions, the PD-1/PD-L1 pathway modulates the immune response by downregulating T cells, preventing auto-immunity.^1^^,^^2^ Cancer cells utilize this pathway to evade a cytotoxic T-cell-mediated immune response by upregulating PD-L1 expression.^3^ The anti-PD-1 therapy pembrolizumab is now a routine therapeutic option in advanced NSCLC, with improved progression-free and overall survival used alone or in combination with cytotoxic chemotherapy.^4^^,^^5^ PD-L1 expression determined by immunohistochemistry is a validated predictive biomarker for anti-PD-1/PD-L1 response in NSCLC. PD-L1 expression ≥50% is associated with improved response and survival with anti-PD-1/PD-L1 immunotherapy, negating the need for combination with cytotoxic chemotherapy and increased toxicities.^6^

As the PD-1/PD-L1 interaction is also exhibited in normal organs, its inhibition has a direct effect on immune homeostasis with the potential for immune-related adverse events (irAEs).^7^^,^^8^ A meta-analysis of 12,808 oncologic patients treated with anti-PD-1/PD-L1 agents found that the overall incidence of irAEs was 26.82% (95% CI, 21.73-32.61; *I*^2^, 92.80) in any grade and 6.10% (95% CI, 4.85-7.64; *I*^2^, 52.00) in severe grade.^9^ Less common irAEs, such as pneumonitis, myocarditis, myositis, nephritis, and haematologic toxicities, are notable for their potential severity.^10^ The exact mechanism behind these irAEs is unknown, and there are limited data on how anti-PD-1/PD-L1 immunotherapy affects PD-L1 expression in normal tissues.

[^18^F]-fluorodeoxyglucose positron emission tomography/computed tomography ([^18^F]FDG PET/CT) is a commonly adopted imaging technique that evaluates glycolytic metabolism and is routinely used in cancer detection, staging, and monitoring treatment response.^11^^,^^12^ Research has shown how performing [^18^F]FDG PET/CT can be predictive in assessing tumour response and reporting irAEs in patients undergoing immune checkpoint inhibition immunotherapy.^13^^,^^14^ Importantly, studies have also demonstrated a close association between PD-L1 and glucose transporter 1 (GLUT1) expression.^15^ However, the impact of anti-PD-1/PD-L1 therapy on [^18^F]FDG PET/CT uptake in normal tissues is unknown.

NM-01 is a novel single-domain antibody (nanobody) to PD-L1 that, when radiolabelled with [^99m^Tc], can be imaged with single-photon emission computed tomography/computed tomography (SPECT/CT) to determine PD-L1 expression.^16^ An early-phase study has shown that [^99m^Tc]NM-01 is stable and has a high binding affinity specifically for PD-L1, with tumour uptake readily visible against background tissues, demonstrating potential efficacy in measuring PD-L1 expression in NSCLC.^17^ As a result, [^99m^Tc]NM-01 SPECT/CT would potentially be a safer non-invasive alternative to multiple biopsies in determining whole-body PD-L1 expression and detecting intra- and inter-tumoral heterogeneity to improve the predictive value of PD-L1 assessment for directing anti-PD-1/PD-L1 therapy. NM-01 binds specifically to human PD-L1 (*K_d_* = 0.8 nM) and does not interfere with the binding of the anti-PD-L1 antibody atezolizumab.^16^ Within our study, we would not expect pembrolizumab to change the targeting of the imaging agent.

This study aims to evaluate the impact of anti-PD-1 therapy on PD-L1 expression and glucose metabolism in normal organs in patients with advanced NSCLC using a dual SPECT/CT and PET/CT imaging approach. The study hypothesis is that anti-PD-1 therapy alters normal organ PD-L1 expression and glucose metabolism in patients with NSCLC. In this study, we compared baseline and 9-week scans of 10 patients with advanced NSCLC to analyse the effects of anti-PD-1 therapy on PD-L1 expression determined by [^99m^Tc]NM-01 SPECT/CT and glucose metabolism on [^18^F]FDG PET/CT of normal organs.

## Methods

### Participants

Ten subjects with advanced NSCLC (mean age 66.2 ± 6.2 years, range 58-76 years; 7 male) were recruited to the PECan study (NCT04436406) between October 2020 and October 2022.^18^^,^^19^ The study was approved by a UK Research Ethics Committee and Health Research Authority (IRAS reference 256684) and all participants provided written informed consent. Five received the anti-PD-1 pembrolizumab alone and 5 with cytotoxic chemotherapy (carboplatin and pemetrexed). Patient files and clinical results were studied to exclude irAEs during the study period.

### Scans

Both [^99m^Tc]-NM-01-PD-L1 SPECT/CT and [^18^F]FDG PET/CT scans were conducted before and 9 weeks after commencing treatment.

PD-L1 expression was measured on SPECT/CT scans using a Siemens Symbia Intevo Bold SPECT/CT scanner with xSPECT Broad Quantification software (Siemens Healthcare GmBH; Erlangen, Germany). Participants received an intravenous bolus of [^99m^Tc]-NM-01-PD-L1, corresponding to 100 mcg of NM-01 (mean injected activity of 565.6MBq ± 99.3 MBq). Single field of view SPECT/CT imaging focused on the primary tumour and suspected metastatic sites. Scans used low-energy, high-resolution collimators with a ± 10% energy window around 140 keV. A 10% energy window centred at 120 keV was also used for scatter correction. SPECT was performed over 180° with 128 projections at 20 s per frame. A CT (110 kV, 25 mA, CTDI average 5.55 mGy, DLP average 246 mGy.cm) was done for anatomical correlation and attenuation correction. Images were reconstructed within an xSPECT Broad Quantification reconstruction workflow using OSEM iterative reconstruction (2 iterations, 10 subsets) with an additive update mechanism, at a matrix size of 128 × 128, with scatter correction.

[^18^F]FDG PET/CT scanning protocols were followed, including fasting for 6 h and voiding before the examination. Scans occurred 1 hour after injecting [^18^F]FDG (mean injected activity = 341.3 MBq ± 29.7 MBq) with blood glucose measurements of <10mmol/L. Images were acquired from the skull base to the upper thighs using a GE Discovery 710 PET/CT scanner with a 20-min scan duration. A low-dose CT scan (140 kV, 10 mA, 0.5 s rotation time, 40 mm collimation) was performed at the start to provide attenuation correction. The PET data were corrected for dead time, scatter, randoms, and attenuation using standard algorithms provided by the scanner manufacturer. Images were reconstructed using iterative reconstruction with time-of-flight (reconstruction parameters: 2 iterations, 24 subsets, Gaussian postfilter with 6.4 mm full width at half maximum, 4 mm voxels).

### Image analysis

For each scan, the maximum and mean standardized uptake values (SUVmax, SUVmean) were quantified and normalized to the patient’s total body weight and measured on normal organs by drawing regions of interest (ROIs) and volumes of interest (VOIs) to assess PD-L1 expression on SPECT/CT and glucose metabolism on [^18^F]FDG PET/CT.

**Figure 1.**
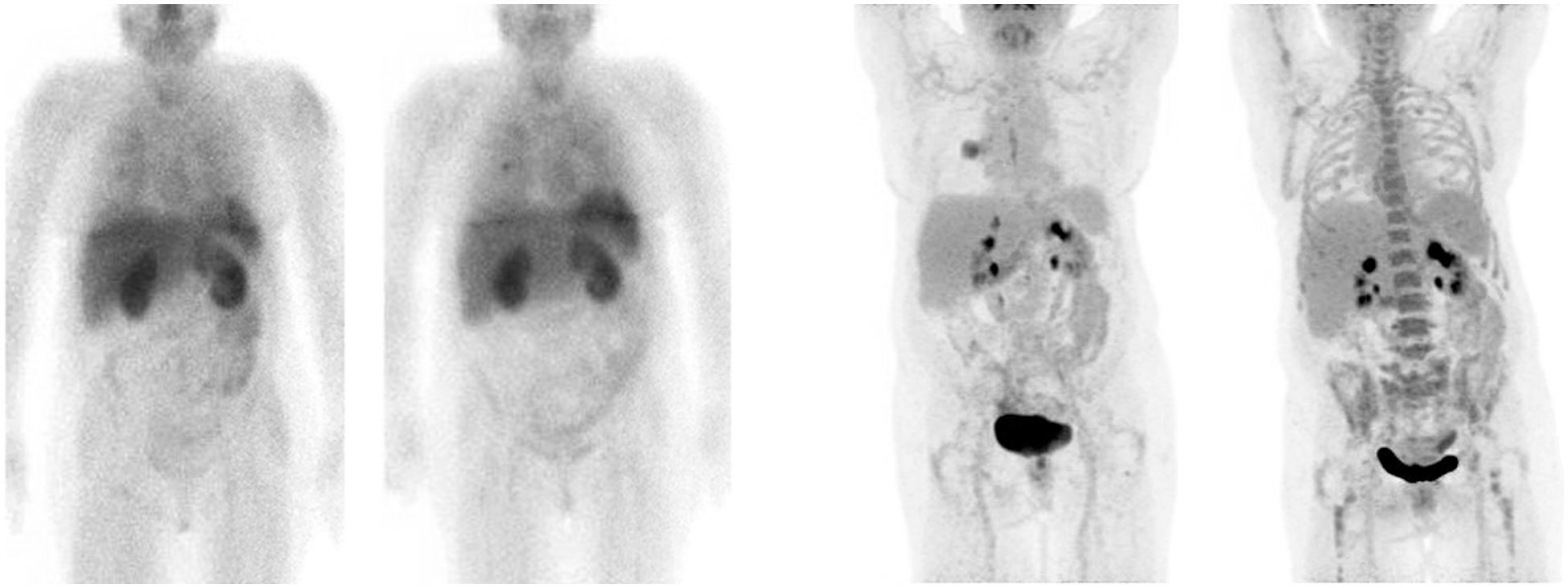
Baseline and follow-up [99mTc]NM-01 (left) and [18F]FDG (right) scans showing NSCLC and bone marrow [18F]FDG uptake changes.

The normal organs measured included blood pool, left ventricular myocardium (LV), lung, liver, spleen, bone marrow, muscle, kidney, and pancreas (Figure 1).

For organs, including the LV and kidney, a freehand technique for the ROI was used. The VOI for other organs was delineated using a uniform sphere. This information is summarized in Table 1.

**Table 1.** ROI/VOI placement details.

Organ	VOI Size	Placement description
Left ventricle (LV)	Freehand ROI	Axial slice through the centre of the LV.
Kidney	Freehand ROI	Right kidney cortex, avoiding areas of system activity.
Blood pool	1.5 cm sphere	At the level of the aortic arch.
Lung	3 cm sphere	At the axial level of the carina.
Liver	3 cm sphere	Halfway down the right lobe of the liver.
Spleen	2 cm sphere	At the level of the gastroesophageal junction (GOJ).
Bone Marrow	1.5 cm sphere	In the centre of the vertebra, at the axial level of the carina.
Muscle	1.5 cm sphere	In the left paravertebral muscle at the level of the midpoint of the LV axial slice.
Pancreas	1 cm sphere	In the centre of the body of the pancreas.

ROI/VOIs were independently placed by 2 observers after training by an experienced researcher.

We directly compared the results obtained by each observer. Inter-observer variation was measured using an intraclass correlation coefficient.

### Statistical analysis

IBM SPSS Statistics (version 28) was used to analyse the data. The Shapiro-Wilk test was used to test for the normality of data distribution, and the *t*-test or Wilcoxon signed-rank test was used accordingly. A *P*-value of <0.05 was considered statistically significant.

## Results

The patient cohort in this study comprised 10 patients diagnosed with advanced non-small cell lung cancer (NSCLC). The group consisted of 7 males with an overall mean age of 66 years. No irAEs were reported during the study.

Five patients received monotherapy with pembrolizumab, a PD-1 monoclonal antibody and 5 were treated with a combination of pembrolizumab and cytotoxic chemotherapy, specifically carboplatin and pemetrexed.

The percentage change in SUVmax and SUVmean between baseline and 9-week scans for both PD-L1 and glucose metabolism is shown in Table 2.

**Table 2.** Changes in PD-L1 expression/glucose metabolism over 9 weeks (mean ± SD; median with range).

			PD-L1 SPECT		^18^F-FDG PET	
	**Organ**	**SUV**	**0-week**	**9-week**	**0-week**	**9-week**
	Blood pool	Max	1.4 ± 0.4	1.3 ± 0.4	2.2 ± 0.4	2.1 ± 0.5
		Mean	1.0 (0.5-1.8)	1.0 (0.6-1.5)	1.7 ± 0.3	1.8 ± 0.5
	Left ventricle (LV)	Max	3.0 ± 0.8	2.9 ± 1.1	2.4 (1.8-8.4)	2.7 (1.7-10.1)
		Mean	2.3 ± 0.5	2.3 ± 0.9	1.6 (1.3-5.7)	1.9 (1.4 -6.9)
	Lung	Max	1.3 (0.7-2.4)	1.0 (0.7-2.2)	0.5 ± 0.2	0.6 ± 0.2
		Mean	0.7 ± 0.3	0.8 ± 0.4	0.3 ± 0.1	0.3 ± 0.1
	Liver	Max	5.2 (3.8-6.5)	5.0 (3.8-8.6)	2.9 ± 0.6	3.0 ± 0.7
		Mean	4.0 ± 0.9	4.0 ± 1.7	2.2 ± 0.4	2.3 ± 0.6
	Spleen	Max	18.9 ± 3.1	20.0 ± 4.3	2.5 ± 0.6	2.5 ± 0.6
		Mean	16.4 ± 3.2	18.0 ± 3.9	2.0 ± 0.6	2.1 ± 0.6
	Muscle	Max	1.0 ± 0.2	0.9 ± 0.3	1.2 (0.9-1.9)	1.2 (0.8 -2.5)
		Mean	0.6 ± 0.2	0.5 ± 0.2	0.8 (0.6-1.4)	0.8 (0.5 -1.7)
	Bone Marrow	Max	3.4 (2.6-5.0)	3.1 (2.5-4.8)	2.6 (1.7-5.5)	2.8 (2.1-6.6)
		Mean	2.4 (1.9-4.5)	2.7 (1.9-4.4)	2.1 (1.3-4.4)	2.3 (1.7-5.5)
	Kidney	Max	48.8 ± 25.0	47.9 ± 20.6	4.0 ± 0.6	4.2 ± 1.0
		Mean	33.4 ± 15.5	33.3 ± 14.6	2.6 ± 0.4	2.7 ± 0.6
	Pancreas	Max	1.8 ± 0.4	1.8 ± 0.3	1.9 ± 0.3	1.7 ± 0.4
		Mean	1.3 ± 0.4	1.3 ± 0.4	1.5 ± 0.2	1.4 ± 0.4

No statistical significance was found in the *P*-values (*P* > .05).

When considering the tracer for PD-L1, NM01, as assessed by SPECT imaging, the uptake values showed no significant changes in PD-L1 expression over the 9 weeks across all organs. For instance, the SUVmax in the spleen showed a non-significant increase from 18.9 ± 3.1 (mean ± SD) at baseline to 20.0 ± 4.3 at 9 weeks (*P* = .36), while the SUVmean also rose from 16.4 ± 3.2 to 18.0 ± 3.9 (*P* = .14). Conversely, the liver had a non-significant decrease in SUVmax from 5.2 to 5.0 (*P* = .65) and showed no significant change in SUVmean. The kidney demonstrated the highest uptake at baseline, possibly due to the role of PD-L1 in inhibiting immunopathology in the kidney,^20^ with an SUVmax of 48.8 ± 25.0 at baseline, which showed a non-significant decrease to 47.9 ± 20.6 at 9 weeks (*P* = .86).

For glucose metabolism, as measured by [^18^F]FDG PET/CT, the uptake was notably stable within the same organ tissue, with only minor differences between organ tissues. For example, the blood pool exhibited a non-significant increase in SUVmean from 1.7 ± 0.3 to 1.8 ± 0.5 (*P* = .51) from 0-week to 9-week. The lung showed a consistent mean uptake, remaining at 0.3 ± 0.1 to 0.3 ± 0.1 (*P* = .34) in the same time frame. On the other hand, the liver showed a non-significant increase in SUVmax from 2.9 ± 0.6 to 3.0 ± 0.7 (*P* = .60) and a non-significant increase in SUVmean. The pancreas displayed a non-significant decrease in SUVmax from 1.9 ± 0.4 to 1.7 ± 0.4 (*P* = .33) from 0-week to 9-week, and a non-significant decrease in SUVmean.

Although some individual patients showed changes in PD-L1 expression and [^18^F]FDG levels, overall, no significant statistical differences (*P* > .05) were observed in the results. The treatment regimen (combined therapy vs anti-PD-1 immunotherapy only) did not significantly change normal organ PD-L1 expression and glucose metabolism.

There was excellent reliability of measurements between the 2 observers with an intraclass correlation coefficient of 0.996 (95% CI, 0.995-0.997; *P* < .001).

## Discussion

Our study presented preliminary data regarding PD-L1 expression measured by [^99m^Tc]NM01 SPECT/CT and glucose metabolism measured by [^18^F]FDG PET/CT in normal organs of patients with advanced NSCLC undergoing anti-PD-1 immunotherapy ± chemotherapy. For PD-L1 expression and glucose metabolism, the results (Table 2) showed no significant difference for SUVmax and SUVmean between baseline and 9-week scans after anti-PD-1 immunotherapy. Instead, we observed stability in both markers in this small cohort with high interobserver reliability. No irAEs were reported during the study; therefore, it is possible that patients with irAEs might show different results.

There is little in the scientific literature describing the expression of PD-L1 within normal tissues. Preclinical data are available that implicate myocardial PD-L1 expression in myocarditis. However, no direct cause has been established.^21^^,^^22^ Within our data set, one patient undergoing both chemotherapy and anti-PD-1 immunotherapy demonstrated increased glucose metabolism across all organs (SUVmax range +1.5% in the liver to +69.6% in the left ventricle myocardium), hinting at potential subclinical immune inflammation from treatment, although no clinical irAE symptoms were experienced. However, as this observation was limited to one patient, it is not possible to draw definitive conclusions.

An endocrinological study investigated 49 tissues, including pancreatic tissue, to establish the cause of endocrinopathies found in anti-PD-1 therapy. The study findings suggested no role in PD-L1 expression in the pathogenesis of endocrine irAEs.^23^ Our results agreed with no significant changes shown in PD-L1 and glucose metabolism in the pancreas.

Additionally, 7 out of 10 of our cohort (Table 3) showed a small increase in glucose metabolism in the spleen and bone marrow between the baseline and 9-week PET/CT scan interval (3 chemotherapy + anti-PD-1 immunotherapy and 4 anti-PD-1 immunotherapy only), suggesting increased immune response, but overall results were not statistically significant. Furthermore, there is a gap in the literature regarding the specific adverse effects on the spleen in anti-PD-1 immunotherapy.

**Table 3.** Percentage change in ^18^F-FDG (PET) for patients with combined chemotherapy and anti-PD-1 immunotherapy, and anti-PD-1 immunotherapy alone over 9 weeks.

18F-FDG (PET)			18F-FDG (PET)
Chemotherapy + anti-PD-1 immunotherapy			Anti-PD-1 immunotherapy alone
% change			% change
Organ	SUV	Group A	Group B
Blood pool	Max	Range: −32.3 to +14.6	Range: −12.5 to +31.5
	Mean	Range: −29.3 to +19.0	Range: −3.5 to +33.8
Left ventricle (LV)	Max	Range: −3.4 to +357.9	Range: −70.6 to +19.8
	Mean	Range: −11.0 to +344.9	Range: −73.1 to +27.7
Lung	Max	Range: −17.1 to +25	Range: −21.2 to +30.4
	Mean	Range: −25.0 to +7.9	Range: −17.2 to +66.7
Liver	Max	Range: −19.3 to +2.1	Range: +3.0 to +21.4
	Mean	Range: −21.7 to +18.0	Range: +5.2 to +28.6
Spleen	Max	Range: −24.1 to +11.5	Range: −4.9 to +68.1
	Mean	Range: −29.8 to +22.5	Range: −3.8 to +98.7
Muscle	Max	Range: −23.2 to +33.9	Range: −20.2 to +14.9
	Mean	Range: −18.5 to +29.2	Range: −18.1 to +7.0
Bone marrow	Max	Range: −20.5 to +130.2	Range: −32.5 to +62.1
	Mean	Range: −21.3 to +133.0	Range: −29.2 to +70.3
Kidney	Max	Range: −10.2 to +36.3	Range: −39.2 to +27.6
	Mean	Range: −8.9 to +15.9	Range: −13.5 to +8.0
Pancreas	Max	Range: −31.3 to +36.9	Range: −27.1 to +22.8

Of note, all patients receiving only anti-PD-1 immunotherapy (Table 4) had a non-statistically significant increase of [^18^F]FDG in liver SUVmax and SUVmean, hinting at potential immune-related changes in hepatic metabolism or subclinical inflammation, an irAE associated with anti-PD-1 monotherapy.^10^ The small cohort size and varied treatments (some receiving both chemotherapy and immunotherapy, others only immunotherapy) may have impacted the results. Future studies could consider only patients undergoing immunotherapy to isolate the effects of chemotherapy as in our study it is not possible to conclude whether small observed effects were due to chemotherapy or immunotherapy in those patients on combined treatment. For example, chemotherapy may alter [^18^F]FDG uptake in the bone marrow.^24^ Future studies could incorporate a larger sample size to increase the possibility of detecting small changes and patients who had experienced irAEs in normal organs, thus leading to a better understanding of the mechanisms behind how immunotherapy can cause irAEs and better screening tools. The occurrence of irAEs could indicate that the immune system has been activated to fight against the malignancy, which can serve as a predictive marker for the response to anti-PD-1 immunotherapy.^25^ However, this is not a strong correlation, warranting further investigation in future studies.

**Table 4.** Percentage change in PD-L1 (SPECT) expression for patients with combined chemotherapy and anti-PD-1 immunotherapy, and anti-PD-1 immunotherapy alone over 9 weeks.

PD-L1 (SPECT)			PD-L1 (SPECT)
Chemotherapy + anti-PD-1 immunotherapy % change			Anti-PD-1 immunotherapy alone% chang
	
Organ	SUV	Group A	Group B
Blood Pool	Max	Range: −39.8 to +32.6	Range: −15.7 to +37.8
	Mean	Range: −47.7 to +32.9	Range: −16.9 to +54.0
Left Ventricle (LV)	Max	Range: −33.9 to +23.8	Range: −29.8 to +18.4
	Mean	Range: −34.6 to +31.6	Range: −34.5 to +20.7
Lung	Max	Range: −28.4 to +9.9	Range: −39.8 to +13.0
	Mean	Range: −17.9 to +41.1	Range: −35.1 to +16.8
Liver	Max	Range: −29.9 to +9.2	Range: −13.0 to +40.8
	Mean	Range: −26.6 to +27.1	Range: −14.9 to +56.5
Spleen	Max	Range: −21.9 to +29.4	Range: −17.7 to +22.4
	Mean	Range: −21.4 to +35.3	Range: −10.8 to +33.3
Muscle	Max	Range: −37.3 to −3.0	Range: −28.3 to +35.6
	Mean	Range: −45.0 to +13.5	Range: −11.1 to +20.4
Bone Marrow	Max	Range: −27.7 to +62.7	Range: −36.8 to +44.9
	Mean	Range: −15.1 to +70.5	Range: −38.3 to +43.6
Kidney	Max	Range: −38.3 to +77.0	Range: −31.4 to +54.6
	Mean	Range: −32.3 to +34.9	Range: −35.1 to +63.9
Pancreas	Max	Range: −29.3 to +21.4	Range: −7.2 to +13.9

It is also pertinent to mention that both [^99m^Tc]NM-01 SPECT/CT and [^18^F]FDG PET/CT scans were performed in a 9-week interval after participants were administered anti-PD-L1 ± chemotherapy drugs. It may take several treatment cycles before a significant effect can be observed although immune-related adverse events commonly occur between 2.2 and 14.8 weeks.^26^ Future studies could extend for a longer duration, with interim scans to be conducted between the study period for a more accurate comparison of results.

Immunotherapeutic drugs have transformed treatments for multiple types of cancers. However, there is limited evaluation of the impact of immunotherapeutic drugs on normal organs in patients with other cancers that express PD-L1, such as melanoma, bladder cancer and renal cell carcinoma.^27^ For this reason, future studies can also be widened to include those patients with other malignancies that can be treated with immunotherapy of variable types. Further study on these areas is essential for earlier and more sensitive detection of irAEs, facilitating the development of future immunotherapies and advancing our understanding of targeting the PD-1/PD-L1 pathway in cancer treatment.

## Conclusions

While no statistically significant effect on normal organs was observed, the stability of glucose metabolism and PD-L1 expression remains an interesting finding. Our results show that [^18^F]FDG uptake did not change with anti-PD-1 therapy, increasing confidence that changes in [^18^F]FDG on anti-PD-1 immunotherapy may be related to other aetiology, including immune-related adverse events. There is no significant change in PD-L1 expression in normal organs following anti-PD-1 therapy, increasing confidence that changes in [^99m^Tc]NM-01 uptake of tumour/metastasis are related to changes in PD-L1 within the tumour.

More research is required on the impact of anti-PD-1 immunotherapy drug combinations to investigate their effects on normal organs and the mechanism behind the resulting irAEs.
